# A Case of Chorioretinitis with Retinal Angiomatous Proliferation

**DOI:** 10.1155/2021/3564939

**Published:** 2021-12-26

**Authors:** Yanru Chen, Mingyan Wei, Qian Chen, Minghan Li

**Affiliations:** ^1^Xiamen University Affiliated Xiamen Eye Center, Xiamen, Fujian 36100, China; ^2^Fujian Key Laboratory of Ocular Surface and Corneal Diseases, Xiamen, Fujian 36100, China; ^3^Department of Ophthalmology, Xiang'an Hospital of Xiamen University, Xiamen, Fujian 36102, China; ^4^Fujian Provincial Key Laboratory of Ophthalmology and Visual Science, Xiamen, Fujian 36102, China; ^5^Eye Institute of Xiamen University, Xiamen, Fujian 36102, China; ^6^School of Medicine, Xiamen University, Xiamen, Fujian 36102, China

## Abstract

A 48-year-old woman had an acute blurred vision in the right eye immediately after drainage of liver abscess. Her best corrected visual acuity (BCVA) was 8/400; fundus photography suggested the diagnosis of endogenous endophthalmitis with chorioretinitis and vitritis. Due to the bad systemic condition, a systemic antibiotic combined with periocular triamcinolone (TA) was carried out first. Inflammatory cells in the vitreous cavity were decreased after treatment; however, fundus fluorescein angiography (FFA) showed abnormal dilation and leakage of the capillaries and retinal-choroidal anastomose, supporting that there was retinal angiomatous proliferation (RAP). Vitreous interleukin-6 (IL-6) was only slightly elevated; the ratio of interleukin-10 (IL-10) and IL-6 was less than 1, and the etiological test was negative. After receiving intravitreal vancomycin injection combined with periocular TA injection, the patient's BCVA was improved from 16/400 to 20/400 with a reduction in vitreous inflammatory cells. However, the patient's RAP was progressed and her BCVA was dramatically decreased to count finger/30 cm. After intravitreal injection of ranibizumab, the patient's BCVA was 5/400 with a significant shrink in lesions and absorption of hemorrhage, exudation, and fluid. Thus, we suggest that early anti-inflammatory treatment in conjunction with anti-VEGF may achieve a better prognosis in patients with inflammatory retinal angiomatous proliferation (RAP).

## 1. Introduction

Chorioretinitis is a type of posterior uveitis. According to the etiology, it can be divided into noninfectious and infectious chorioretinitis [1]. Early recognition with prompt treatment is fundamental to prevent severe loss of vision. Infectious chorioretinitis can be caused by blood-borne pathogens, such as fungi, viruses, bacteria, and endogenous parasites; endophthalmitis can be the reason [2]. Klebsiella pneumoniae is the main pathogen of liver abscess [3]. Among the extrahepatic invasive manifestations, organs such as the lung, brain, and eye are the most common invasive sites [4]. Pathogenic detection is the gold standard for diagnosis, but medical history and empirical therapy are also important when the diagnosis is ambiguous. Early combination therapy has great value for the patients who have endophthalmitis along with retinal angiomatous proliferation (RAP).

## 2. Case Report

**Figure 1:**
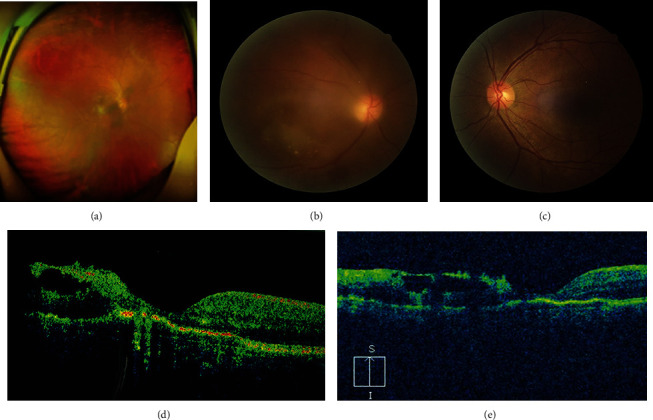
Inflammation was present in the right eye. (a) The fundus was opaque, and exudation and hemorrhage could be seen in the macular and inferonasal peripheral retina. (b) Dense vitreous opacities obscured the visualization of the fundus of the right eye. Chorioretinitis with exudation and hemorrhage was seen in the macular. (c) Normal fundus of left eye. (d, e) OCT showed an epiretinal membrane above the inner retinal surface of macular fovea and pigment epithelial detachment (PED).

A 48-year-old female complained of defective vision in the right eye immediately after drainage of liver abscess. Her best corrected visual acuity was 8/400 in the right eye and 20/20 in the left eye. There was no evidence of anterior chamber cells or keratic precipitates, and the intraocular pressure was normal. Dense vitreous opacities obscured the visualization of fundus of the right eye. Chorioretinitis with exudation and hemorrhage were present in the macular and inferonasal peripheral retina (Figures 1(a) and 1(b)). The fundus of the left eye is normal (Figure 1(c)). Optical coherence tomography (OCT) showed epiretinal membrane on the inner retinal surface of macular foveal and pigment epithelial detachment (PED) (Figures 1(d) and 1(e)). The patient's blood culture was positive for Klebsiella pneumoniae. Rheumatoid factor (RF), antistreptolysin antibody (ASO), human immunodeficiency virus (HIV), TP particle agglutination assay, and purified protein derivative (PPD) tuberculin skin test were all negative. The possibility of endogenous endophthalmitis was considered in ophthalmologic consultation. Taking into account the patient's poor general condition, systemic cephalosporin therapy combined with periocular triamcinolone injection was given to her.

**Figure 2:**
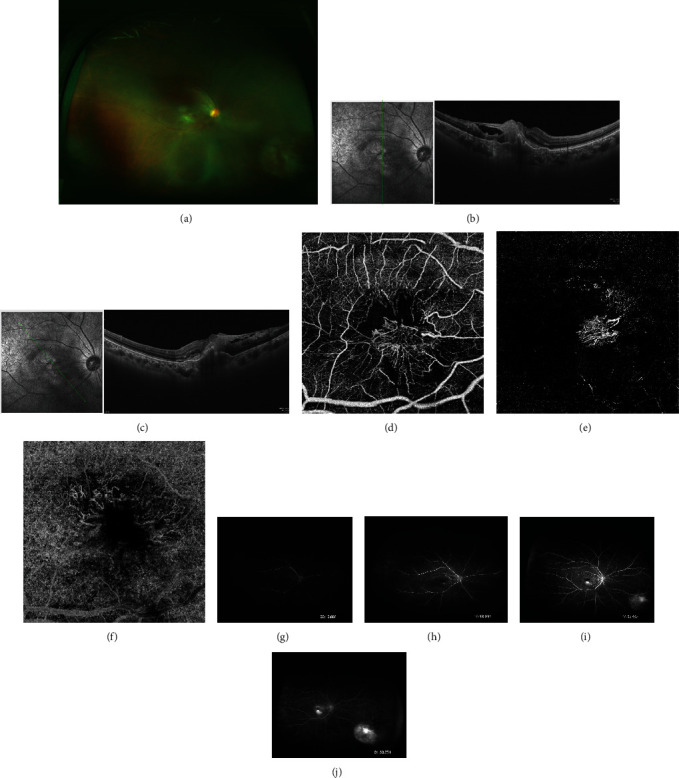
Infectious chorioretinitis and RAP were identified in the right eye. (a) The crooked retinal arterioles and retinal venules extend into deeper layers in the macula. (b, c) OCT showed a thickened epiretinal membrane, hyperreflective material located at the level of the outer retinal layers, and vascularized PED in macular fovea of the right eye. (d–f) OCTA detected abnormal vascular networks in the superficial retinal capillary plexus (d), deep retinal capillary plexus (e), and choroid layers (f). (g–j) Fundus fluorescein angiography (FFA) of the patient: (g) a central, intraretinal hyperfluorescent lesion was visible, which is probably fed by the retinal arteriolar vessel at 2 o'clock; (h) progressive filling of the venous circulation, connected to the hyperfluorescent lesion; (i) the central, intraretinal vascular complex was clearly recognizable in macular, and a round hyperfluorescent lesion was detected at inferonasal peripheral retina; (j) leakage of fluorescent dye from the vascular complex, abnormal dilation of the capillaries, and the hyperfluorescent lesion.

Four months later, the patient returned to the ophthalmology department with stable condition after the antibiotic therapy. Her BCVA was 16/400 and vitreous inflammatory cells in the right eye were decreased; flaky yellowish-white elevated lesions surrounded by hemorrhages can be visualized in the macular and inferonasal peripheral retina. The crooked retinal arterioles and retinal venules extended into the outer nuclear layers of the macula of the right eye (Figure 2(a)). OCT showed that a dense epiretinal membrane was attached in the inner surface of the retina, hyperreflective materials were located at the level of the outer retinal layers, and vascularized PED was found in the macular fovea of the right eye (Figures 2(b) and 2(c)). Optical coherence tomography angiography (OCTA) detected abnormal vascular networks in the superficial, deep retinal capillary plexus/inner retina, outer retina, and choroidal layers (Figures 2(d)–2(f)). Fundus fluorescein angiography (FFA) showed that an intraretinal vascular complex was formed by retinal artery and retinal vein anastomosis in the macular, and a hyperfluorescent lesion was detected at the inferonasal peripheral retina of the right eye (Figures 2(g)–2(j)). During the course of the fluorescein angiography, the vascular complex, abnormal dilation of the capillaries, and hyperfluorescent lesion had progressively increased levels of leaked dye. Vitreous humor testing was performed, and the results showed that interleukin-6 (IL-6) was 33 mg/ml, which was slightly higher than normal, suggesting that intraocular inflammation was present. However, polymerase chain reaction (PCR) showed that the pathogen was negative. The ratio of interleukin-10 (IL-10) and IL-6 was less than 1, indicating that the diagnosis of lymphoma was not supportive. Based on the symptoms and clinic tests, the clinical diagnosis was endophthalmitis with RAP. Considering that the patient had an infection history and presented chorioretinitis, empirical treatments such as intravitreal vancomycin injection combined with periocular triamcinolone injection were recommended. After treatment, her vitreous inflammatory cells of the right eye were decreased; the patient's BCVA was improved to 20/400.

**Figure 3:**
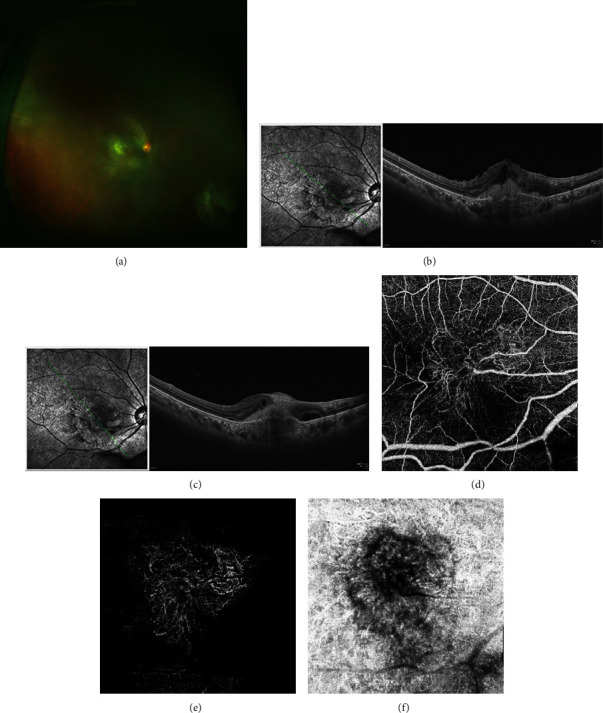
The deterioration of RAP in the right eye. (a) Inflammatory cells in the vitreous cavity disappeared and fundus can be seen clearly. (b) The central retinal thickness increased; intra- and subretinal fluid produced. (c) OCTA showed the area of abnormal vascular network in the inner retina (d) and outer retina (e), and choroid layers (f) were enlarged.

**Figure 4:**
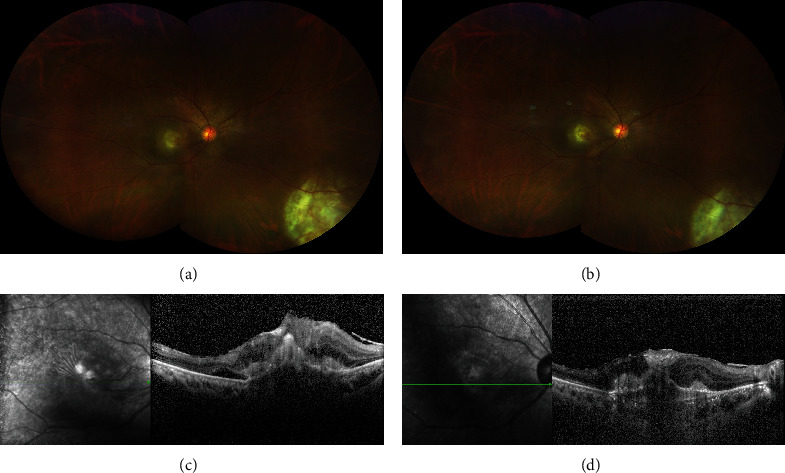
Comparation of lesions before and after the treatment of anti-VEGF. (a, b) The color fundus photograph of the right eye before and after treatment. (a) The lesions have obscure boundaries and are surrounded with hemorrhages. (b) The yellow-white lesions in the macular and inferonasal peripheral retina. Retina were well-demarcated and shrunk, hemorrhage was absorbed, and exudation around the optic nerve reduced significantly. (c, d) The OCT images of RAP before and after treatment: (c) intra- and subretinal fluids were increased; (d) sub- and intraretinal fluids were partly absorbed, and central retinal thickness was reduced from 841 *μ*m to 690 *μ*m.

Five months later, the inflammation was under control (Figure 3(a)), but her RAP was progressed and resulted in serious damage in her central vision. Her BCVA was decreased to count finger (CF/30 cm). OCT showed that intra- and subretinal fluids were increased (Figures 3(b) and 3(c)). Areas of abnormal vascular network on OCTA were enlarged (Figures 3(d)–3(f)). Anti-VEGF therapy was given to her. After three intravitreal injections of ranibizumab (once a month), the lesions shrunk compared to before the VEGF treatment (Figures 4(a) and 4(b)). OCT revealed that sub- and intraretinal fluids were partly absorbed than before in the right eye (Figures 4(c) and 4(d)). The patient's BCVA was improved to 5/400.

## 3. Discussion

In this case, the patient had blurred vision immediately after drainage of liver abscess; Klebsiella pneumoniae was positive in blood culture, so endogenous infectious endophthalmitis was assumed at the initial diagnosis. Considering the poor systemic condition of the patient, systemic antibiotic therapy combined with periocular TA was carried out first [5]. Four months later, the patient's systemic condition was stable, and inflammatory cells in the vitreous were decreased. However, FFA showed abnormal dilation and leakage of the capillaries, suggesting that inflammatory activity was still present. Vitreous humor testing showed that IL-6 was only slightly elevated, and the etiological test was negative. It may be explained as the following reasons: (1) the patient has received treatments for liver abscess, and the information is under control; (2) although the positive rate of PCR detection can be more than 90% [6–9], there may be still false negatives; (3) the patient actually has noninfectious chorioretinitis due to the hypoimmunity caused by liver abscess and surgery. Considering that the patient had a confirmed infection history, she was treated by the empirical intravitreal injection of vancomycin combined with periocular TA injection [10]. After the treatment, the vitreous inflammatory cells reduced, suggesting the inflammation was controlled, which supported our primary diagnosis as infectious endophthalmitis.

In inflammatory eye diseases, inflammatory factors promote the upregulation of vascular endothelial growth factor (VEGF), accelerate the development of abnormal angiogenesis, and often result in concurrent inflammatory CNV [11, 12]. RAP is a special subtype of neovascular lesion occurring in age-related macular degeneration (AMD), which is also referred to as type 3 neovascularization. Although its origin is controversial [13–16], it is characterized by detecting chorioretinal anastomosis as neovascularization. Chorioretinitis with RAP is not common, and attention should be paid to distinguish it from inflammatory CNV. In this patient, FFA showed large areas of capillary dilation; leakages of fluorescent dye were present in macular and inferonasal peripheral retinal lesions, retinal arterioles and venules extended into the avascular photoreceptor layers, and the choroid vessels also extended through the disrupted retinal pigment epithelial, suggesting the formation of retinal-choroidal anastomoses (RCA). OCTA detected abnormal vascular network in superficial, deep retina, and choroidal layers. All of the evidence supported the diagnosis of chorioretinitis and RAP.

Most of documented studies have recommended the application of intravitreal injection of anti-VEGF drug for treating RAP, which was considered as a form of exudative AMD [17–19]. Since inflammation was involved in the pathogenesis of RAP, recent research studies have shown the efficacy of intravitreal or periocular TA injection in the treatment of RAP [20, 21]. In this case, the patient only received antibiotic and triamcinolone therapy in the early stage due to the severe systemic inflammatory reactions. Several months later, her inflammation was under control, but her RAP was progressed and her visual function suffered serious damage. Anti-VEGF drugs can reduce inflammation, which has been widely demonstrated in studies of inflammatory CNV [22, 23], and has a high efficacy in RAP with uveitis. In a case report, Haq et al. demonstrated a successful treatment for RAP with combined intravitreal ranibizumab and intravitreal TA [24]. After combined treatment with intravitreal ranibizumab, the fundus lesions significantly shrunk, and the hemorrhage, exudation, and fluid significantly were absorbed, suggesting that the anti-VEGF treatment was effective. Therefore, we concluded that early anti-inflammatory treatment combined with anti-VEGF may achieve a better prognosis in patients with RAP and inflammation.

## 4. Conclusion

RAP with endophthalmitis is rare; early anti-inflammatory and anti-VEGF treatments were effective in this case. When vitreous humor and laboratory results are ambiguous to distinguish infectious from noninfectious inflammation, the history and clinical signs may help to support the primary diagnosis and experimental treatment.

## Data Availability

The data are available in the article or on request.
